# Serum leptin and serum leptin/serum leptin receptor ratio imbalance in obese rheumatoid arthritis patients positive for anti-cyclic citrullinated peptide antibodies

**DOI:** 10.1186/s13075-015-0850-8

**Published:** 2015-11-20

**Authors:** Eduardo Gómez-Bañuelos, Rosa Elena Navarro-Hernández, Fernanda Corona-Meraz, Perla Monserrat Madrigal-Ruíz, Beatríz Teresita Martín-Marquez, Oscar Enrique Pizano-Martinez, Jorge Aguilar-Arreola, Paul Jacob Perez-Cruz, Hector Macias-Reyes, Laura Gonzalez-Lopez, Jorge Ivan Gamez-Nava, Mario Salazar-Páramo, Monica Vazquez-del Mercado

**Affiliations:** Instituto de Investigación en Reumatología y del Sistema Musculoesquelético, CUCS, Universidad de Guadalajara, Sierra Mojada No. 950, Colonia Independencia, Zip code 44340 Guadalajara, Jalisco México; Servicio de Reumatología, División de Medicina Interna, OPD Hospital Civil de Guadalajara, “Dr. Juan I. Menchaca”, Salvador de Quevedo y Zubieta No. 750, Zip code 44100 Guadalajara, Jalisco >México; Departamento de Medicina Interna-Reumatología, Hospital General Regional no.110, Instituto Mexicano del Seguro Social, Circunvalación Oblatos No. 2212, Colonia Oblatos, Zip code 44700 Guadalajara, Jalisco México; Unidad Médica de Alta Especialidad, Centro Médico Nacional de Occidente, Instituto Mexicano del Seguro Social, Belisario Domínguez No. 1000, Independencia Oriente, Zip code 44340 Guadalajara, Jalisco México

**Keywords:** Leptin, Soluble leptin receptor, Obesity, Rheumatoid arthritis, Anti-CCP antibodies, Leptin/soluble leptin receptor ratio

## Abstract

**Introduction:**

Leptin has a prominent role in the development and maintenance of acute and chronic inflammatory states such as rheumatoid arthritis (RA) and obesity. Nevertheless, the association of serum leptin (sLep) and soluble leptin receptor (sLepR) in RA pathogenesis has not been clarified. The purpose of this study was to evaluate the association of sLep, sLepR and leptin production indexes such as sLep/fat mass ratio with clinical activity and biomarkers and anti-cyclic citrullinated peptide (anti-CCP) antibodies in RA compared with body mass index (BMI) matched control subjects.

**Methods:**

We included 64 RA patients and 66 controls matched for age, gender and BMI. Subjects were evaluated for BMI, fat mass distribution, sLep, sLepR, sLep/fat mass ratio and sLepR/fat mass ratio. Patients were evaluated for clinical activity and anti-CCP antibodies.

**Results:**

We found two or three fold increased sLep levels, sLep/sLepR ratio and sLep/fat mass ratio in obese anti-CCP positive RA patients *vs*. controls. Partial correlations showed that anti-CCP antibodies were correlated with sLep/fat mass ratio (partial r = 0.347, *P* = 0.033) after adjustment for age, subcutaneous adipose tissue and fat mass.

**Conclusions:**

In preobese and obese RA patients there is and increased production of sLep according to anti-CCP positivity. This phenomenon suggests there is an additive effect of chronic inflammation resulting from RA and obesity in which leptin favors the humoral response against citrullinated proteins. In summary, the data observed in our study suggests sLep could be a surrogate marker of chronicity and humoral immunity in RA in the presence of obesity.

## Introduction

Rheumatoid arthritis (RA) is a chronic inflammatory autoimmune disease characterized by joint destruction and disability [1]. The role of proinflammatory cytokines is well-defined in the mechanism of disease in local damage such as pannus formation and bone erosion [1]. In addition, proinflammatory cytokines might contribute to the development of metabolic dysfunction in RA [2]. Adipokines have gained a prominent role within the last decade in the understanding of pathogenesis of chronic diseases (RA, obesity, metabolic syndrome and type 2 diabetes mellitus) [1, 3], through the maintenance of chronic inflammation within the joints or systemically [4]. From the adipokines described so far, the role of leptin is prominent.

Leptin is a non-glycosylated 16 kDa protein from the type I cytokine superfamily that is mainly produced by adipose tissue [5, 6]. Besides the biological function of leptin in food intake and energy expenditure, its role in immune regulation has been acknowledged due to its effects in both innate and adaptive immunity [4, 7]. Leptin signaling is transduced by leptin receptor (LepR), a transmembrane protein of 170 kDa that belongs to the family of type 1 cytokine receptors. Five isoforms of LepR in humans have been described; the isoforms “a” through “d”, are transmembrane proteins obtained by alternative splicing, while, isoform “e” corresponds to soluble leptin receptor (sLepR) obtained by proteolytic shedding of transmembrane isoforms [8].

sLepR could regulate leptin action through high affinity binding of free leptin, preventing its degradation and clearance, but also by avoiding leptin binding to transmembrane receptors and activation of signaling through the signal transducer and activator of transcription 3 protein (STAT3) [9–11]. Increased expression of sLepR is associated with lower body weight in murine models, suggesting the important role of sLepR in leptin regulation [12]. Reduction of sLepR levels occur in progression of obesity with fat mass accumulation, turns into more bioactive leptin for signaling through transmembrane receptors [13–15]. Chronically high levels of leptin may provoke reduced LepR signaling due to a mechanism that is not yet completely elucidated, leading to leptin resistance [16, 17].

Despite the extensive study of leptin in RA there is still no consensus on the role of this molecule in RA pathogenesis. Previous studies included small numbers of patients without a clear cutoff for body mass index (BMI), resulting in non-reproducible associations. In general, serum levels of leptin are more elevated in RA patients than controls and are correlated with body fat mass and BMI [18–20]. Leptin levels are lower in synovial fluid than in plasma and correlate with intra-articular white blood cell count suggesting *in situ* consumption [21]. Moreover, in vitro leptin may induce production of interleukin (IL)-6 in fibroblast-like synoviocytes and IL-8 via janus kinase (JAK)2-STAT3 signaling [22, 23]. In vivo, leptin has been correlated with erythrocyte sedimentation rate (ESR), C-reactive protein (CRP), change in disease activity score in 28 joints (DAS28), erosive disease and progression in the Sharp/van der Heijde score [24–27].

In this study, we evaluated RA patients without traditional cardiovascular risk factors classified by anti-cyclic citrullinated peptide (anti-CCP) positivity, measuring serum leptin (sLep), sLepR, sLep/sLepR ratio and other metabolic parameters, namely proinflammatory cytokines, BMI and adiposity.

## Methods

### Study groups

We included patients with RA attending the rheumatology service of Hospital Civil “Dr. Juan I. Menchaca”, Guadalajara, Jalisco, Mexico. To be eligible for the study, patients had to be 18 years of age or older and meet the American College of Rheumatology criteria (ACR 1987) [28]. For the control group we included volunteers from the open population, who were matched by age, gender and BMI with the RA subjects. We excluded subjects with: previous history of smoking or who were current smokers; cardiovascular disease (CVD); hypertension; diabetes mellitus type 2; thyroid disease; renal impairment; malignancy; hepatic disease or hyperlipidemia. We also excluded patients previously treated with high doses of steroids (>10 mg/day prednisone or equivalent, including those given by intravenous administration).

### Ethics

This protocol was approved by the Institutional Review Board (IRB) committee of “Hospital Civil Juan I. Menchaca”, registered under the number 1068/10. Written consent was obtained from all subjects who participated in the study. Research was conducted according to Declaration of Helsinki.

### Subject assessment

Height was measured using a stadiometer (Seca GmbH & Co. KG. Hamburg, Germany) to the nearest 1.0 mm. Body weight (to the nearest 0.01 kg), BMI and fat mass were determined by bio-electrical impedance analysis (Tanita BC418® Tokyo, Japan). All subjects were classified by BMI according to World Health Organization (WHO) criteria as: normal weight (BMI 18.50–24.99 kg/m^2^), preobese (BMI 25.00–29.99 kg/m^2^) or obese (BMI ≥30.00 kg/m^2^) [29]. Sagittal abdominal diameter (SAD) and waist and hip circumference were measured to the nearest 0.1 cm using an anthropometric fiberglass tape, Gulick® length 0–180 cm precision ±1 mm (North Coast Medical Inc., Gilroy, CA, USA) in accordance with the procedures recommended by Durnnin [30]. Four measures (in millimeters) of skinfold thickness (biceps, triceps, subscapular and supra-iliac) were obtained on the left side of the body using a Harpenden skinfold caliper (opened 80 mm and precision of ± 0.2 mm, constant pressure 10 g/mm^2^; Holtain Ltd. Crosswell, Crymych, UK.) and following the procedures recommended by the anthropometric indicators measurement guide.

We calculated the waist to hip ratio (WHR), body fat ratio:

$$ \left(\mathrm{B}\mathrm{F}\mathrm{R} = \mathrm{Body}\ \mathrm{fat}\ \mathrm{mass}\ \left(\mathrm{kg}\right)/\mathrm{Height}\ \left({\mathrm{m}}^2\right)\right), $$

as an indicator of adiposity, visceral fat area:

$$ \left(\mathrm{V}\mathrm{F}\mathrm{A}=\kern0.5em 6.47\kern0.5em \times \kern0.5em \mathrm{SAD}\kern0.5em +\kern0.5em 186.81 \times \mathrm{W}\mathrm{H}\mathrm{R}\ \hbox{--}\ 10.77 \times \mathrm{Sex}\ \left(\mathrm{man} = 1,\ \mathrm{woman} = 2\right) + 0.94 \times \mathrm{A}\mathrm{ge} + 0.83 \times \mathrm{Body}\ \mathrm{mass}\ \left(\mathrm{kg}\right)\ \hbox{--}\ 290.31\right) $$

[31], 

as an indicator of preferential accumulation of fat in the abdomen rather than on the limbs, and the sum of the four skinfold thicknesses (S4ST) as an indicator of subcutaneous fat. Disease activity was measured in RA patients using the DAS28-based CRP and ESR [32].

### Laboratory techniques and procedures

We obtained venous blood samples that were allowed to clot at room temperature and were subsequently centrifuged at 1,500 relative centrifugal force (RCF) (Rotanta 460R, Andreas Hettich GmbH & Co. KG. Germany) for ten minutes. The serum was stored at −70 °C until analysis.

ESR was measured using the Wintrobe method [33]. CRP, rheumatoid factor (RF) and glucose levels were measured by standard techniques (RANDOX Laboratories Limited, Crumlin, UK). Serum insulin (limit of detection 0.399 μΙU/mL, ALPCO Diagnostics, Salem, NH, USA), sLep and sLepR limit of detection 0.4 ng/mL and tumor necrosis factor α (TNFα), limit of detection of 8.43 pg/mL (Enzo Life Sciencies, Inc. New York, NY, USA) and, anti-CCP antibodies, U/mL (Axis-Shield Diagnostics Ltd., Dundee, Scotland), were measured by the ELISA method. We put forward the follow new indexes as indicators of leptin production: 1) sLep (ng/mL)/sLepR (ng/mL), 2) sLep (ng/mL)/fat mass (kg) and 3) sLepR (ng/mL)/fat mass (kg).

Homeostasis model assessment-insulin resistance (HOMA-IR) was calculated [34] and individuals were classified by Stern criteria as: individuals with insulin resistance (IR) when BMI ≥27.5 kg/m^2^ and HOMA-IR ≥3.6 or HOMA-IR >4.65 independent of BMI, and individuals without IR when negative for one of the two conditions [35].

### Statistical analysis

Data were analyzed with the software SPSS v22.0 (SPSS Inc., Chicago, IL, USA). Results are represented as mean ± SD or frequencies, accordingly. Continuous variables were analyzed with Student’s *t* test or the Mann-Whitney *U* test, accordingly. Qualitative variables were analyzed with the chi square (χ^2^) or Fisher’s exact test. The Pearson *r* correlation coefficient was calculated. One-way analysis of variance (ANOVA) with Dunnet’s T3 post hoc test was used to compare quantitative data between groups. Statistical significance was considered when *P* <0.05.

## Results

### Demographic and clinical characteristics

We evaluated 64 RA patients compared with 66 controls matched by age and gender, classified according to BMI. Age, gender, laboratory tests and metabolic parameters are described in Table 1.

**Table 1:** Demographic and clinical characteristics of the groups studied

	Normal weight			Preobese			Obese		
Measurement	Control	RA	*P*	Control	RA	*P*	Control	RA	*P*
	n = 21	n = 21		n = 26	n = 21		n = 19	n = 22	
Age, years	34 ± 11	39 ± 12	0.127	43 ± 12	47 ± 10	0.230	46 ± 9	41 ± 9	0.076
Body weight, kg	60 ± 7	57 ± 6	0.126	68 ± 6	69 ± 5	0.578	91 ± 18	82 ± 9	0.060
BMI, kg/m^2^	22.00 ± 1.78	22.13 ± 2.27	0.845	27.26 ± 1.16	27.50 ± 1.28	0.498	35.03 ± 6.79	33.55 ± 2.75	0.384
Body fat mass, %	26.36 ± 5.66	25.95 ± 6.79	0.834	36.20 ± 3.24	36.21 ± 3.21	0.991	38.78 ± 8.19	42.35 ± 5.00	0.095
Body fat mass, kg	15.86 ± 3.87	14.67 ± 4.29	0.353	24.54 ± 4.84	24.88 ± 3.24	0.784	35.58 ± 12.01	34.81 ± 7.17	0.806
Body fat ratio, kg/m^2^	5.83 ± 1.47	5.80 ± 1.83	0.958	9.94 ± 1.54	9.97 ± 1.15	0.942	13.86 ± 5.25	14.29 ± 2.60	0.739
VFA, cm^2^	46.38 ± 26.58	77.12 ± 43.28	0.021	81.26 ± 23.62	119.52 ± 35.22	<0.001	106.43 ± 30.24	131.83 ± 21.97	0.036
S4ST, mm	52.08 ± 13.67	49.17 ± 14.48	0.513	105.94 ± 20.79	70.56 ± 19.21	<0.001	90.06 ± 16.94	76.34 ± 23.01	0.015
ESR, mm/h	9.76 ± 6.32	19.38 ± 9.82	0.001	21.19 ± 10.08	17.10 ± 12.14	0.213	14.21 ± 10.20	27.33 ± 14.08	<0.001
CRP, mg/L	5.22 ± 2.72	7.66 ± 7.08	0.153	3.56 ± 3.07	8.83 ± 6.67	0.005	8.66 ± 4.70	11.02 ± 9.19	0.112
Glucose mg/dL	94.43 ± 14.03	96.90 ± 14.27	0.574	93.42 ± 27.18	100.80 ± 16.33	0.223	101.37 ± 11.36	98.25 ± 10.81	0.386
Insulin, μΙU/mL	6.97 ± 6.97	14.43 ± 10.82	0.005	9.98 ± 6.56	14.76 ± 16.54	0.268	12.81 ± 8.35	23.16 ± 44.60	0.240
HOMA-IR	1.49 ± 1.16	3.61 ± 2.98	0.003	2.34 ± 1.59	4.34 ± 4.98	0.171	3.28 ± 2.25	5.58 ± 10.92	0.278
Leptin, ng/mL	30.14 ± 20.69	45.98 ± 40.26	0.119	35.48 ± 12.32	121.65 ± 106.35	0.001	79.11 ± 45.20	188.21 ± 100.88	<0.001
sLepR, ng/mL	68.81 ± 24.02	60.02 ± 18.81	0.209	43.98 ± 15.24	51.34 ± 12.51	0.131	44.75 ± 14.91	37.49 ± 9.67	0.096
Leptin/sLepR	0.53 ± .50	0.98 ± 1.40	0.176	0.89 ± 0.41	2.45 ± 2.45	0.033	2.18 ± 1.62	5.67 ± 3.56	0.002
Leptin/FM, ng/mL/kg	1.87 ± 1.21	3.04 ± 1.95	0.027	1.41 ± 0.423	4.99 ± 4.48	0.002	2.18 ± 1.19	5.32 ± 2.67	<0.001
sLepR/FM, ng/mL/kg	4.59 ± 1.89	4.56 ± 2.33	0.964	1.83 ± 0.71	2.12 ± 0.53	0.196	1.49 ± 0.95	1.10 ± 0.41	0.113

Continuous variables are represented by mean ± standard deviation; means were compared with Student’s *t* test. *RA* rheumatoid arthritis, *BMI* body mass index, *VFA* visceral fat area, *S4AP* sum of four skinfold thicknesses (bicipital, tricipital, subscapular and supra-iliac), *ESR* erythrocyte sedimentation rate, *CRP* C-reactive protein, *sLepR* soluble leptin receptor), *FM* fat mass, *HOMA-IR* homeostatic model assessment-insulin resistance

In patients with RA, fat mass distribution was different to that in controls; RA patients had an increased VFA and lower subcutaneous adipose tissue accumulation (S4ST) than controls despite having similar BMI. In addition, we observed a trend for increased basal insulin and HOMA-IR in patients with RA vs. controls (Table 1).

RA patients had higher levels of sLep (115.91 ± 103.81 ng/mL vs*.* 46.33 ± 34.66 ng/mL, *P* <0.001) and higher sLep/fat mass ratio (4.42 ± 1.02 ng/mL/kg vs. 1.79 ± 1.02 ng/mL/kg, *P* <0.001) than controls. There was no difference in serum sLepR between RA patients and controls (2.75 ± 2.08 ng/mL vs. 2.63 ± 1.86 ng/mL, *P* = 0.755), data not shown.

We observed an increase in sLep levels and sLep/sLepR ratio in obese and preobese RA patients in comparison with controls. In addition, sLep levels and sLep/sLepR ratio in RA patients were increased by fourfold to fivefold in obese patients in comparison with normal-weight patients. Patients with RA had more sLep per fat mass than controls, according to the WHO classification (Table 1).

The prevalence of IR, sLep and sLepR levels and sLep/sLepR ratio in RA patients were as follows: obese > preobese > normal weight, while for ESR levels they were obese > preobese RA patients (Table 2).

**Table 2:** Demographic and clinical characteristics of RA patients according to BMI

Measurement	All	Normal weight	Preobese	Obese
		BMI 18.50–24.99 kg/m^2^	BMI 25.00–29.99 kg/m^2^	BMI ≥30.00 kg/m^2^
	n = 64	n = 21	n = 21	n = 22
Female gender, n (%)	60 (93.8)	19 (90.5)	20 (95.2)	21 (95.5)
Time of disease evolution, years	5.34 ± 6.97	5.73 ± 8.95	5.06 ± 4.80	5.23 ± 6.78
Positive anti-CCP, n (%)	46 (71.9)	15 (71.4)	13 (61.9)	18 (81.8)
Anti-CCP, U/Ml	167.20 ± 305.49	179.03 ± 405.53	218.45 ± 338.76	107.00 ± 86.53
Positive RF, n (%)	41 (65.1)	13 (61.9)	15 (71.4)	13 (61.9)
RF, UI/Ml	79.39 ± 51.57	93.03 ± 43.26	79.65 ± 60.09	68.01 ± 47.61
ESR, mm/h	21.27 ± 12.74	19.38 ± 9.82	17.10 ± 12.14	27.33 ± 14.08^b^
CRP, mg/L	9.14 ± 7.71	7.66 ± 7.08	8.83 ± 6.67	11.02 ± 9.19
DAS-28-based CRP	3.40 ± 1.30	2.90 ± 1.20	3.82 ± 1.24	3.53 ± 1.36
TNFα, pg/Ml	57.05 ± 39.87	62.03 ± 34.40	60.93 ± 53.82	48.83 ± 27.48
sLep, ng/Ml	119.70 ± 104.64	45.98 ± 40.26^a^	121.65 ± 106.35	188.21 ± 100.88
sLepR, ng/Ml	50.45 ± 17.25	60.02 ± 18.81^c^	51.34 ± 12.51	37.49 ± 9.67
sLep/sLepR, ng/mL	2.87 ± 3.19	0.98 ± 1.40^a^	2.45 ± 2.45	5.67 ± 3.56
Insulin resistance, n (%)	18 (36.7)	5 (26.3)	5 (35.7)	8 (50.0)

Continuous variables are represented as mean ± standard deviation; qualitative variables are represented as number (%). Groups were compared with one-way analysis of variance and Dunnet’s T3 post hoc test. ^a^Normal weight vs. preobese and obese; ^b^preobese vs. obese; ^c^Normal weight vs. obese. *BMI* body mass index, *anti-CCP* anti-cyclic citrullinated peptide antibodies, *RF* rheumatoid factor, *ESR* erythrocyte sedimention rate, *CRP* C-Reactive protein, *TNF* tumor necrosis factor, *sLep* soluble leptin), *sLepR* soluble leptin receptor, *HOMA-IR* homeostatic model assessment, *DAS-28* disease activity index in 28 joints

**Fig. 1:**
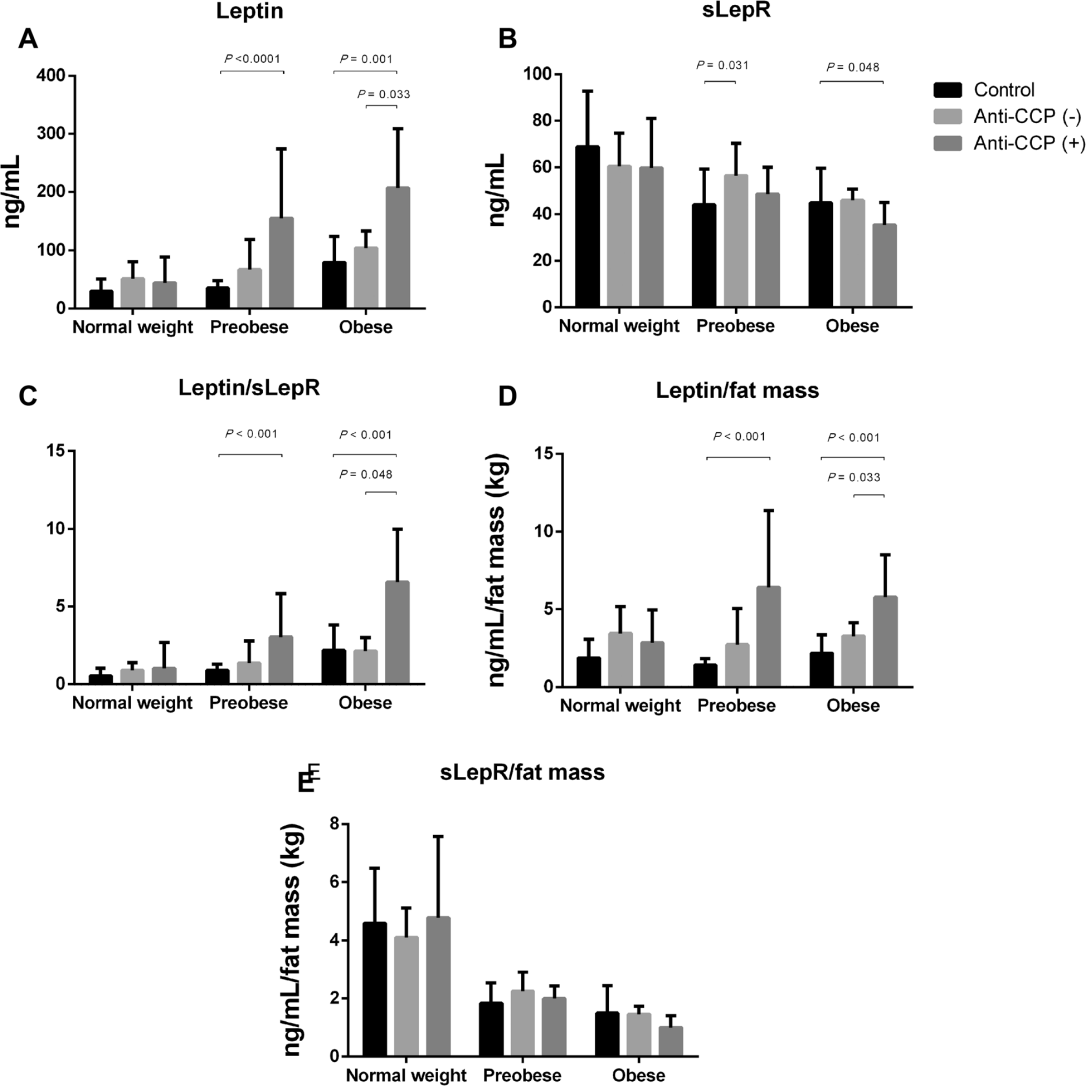
Serum leptin (*sLep*), sLep/sLep receptor (*sLepR*) and leptin/fat mass ratio in controls and patients with rheumatoid arthritis (RA) according to anti-cyclic citrullinated peptide antibodies (*anti-CCP*). Mean ± standard deviation of sLep (**a**), sLepR (**b**), leptin/sLepR (**c**), leptin/fat mass ratio (**d**) and sLepR/fat mass ratio (**e**)

In serum sLep from obese RA patients, the sLep/sLepR ratio and sLep/fat mass ratio were higher according to positivity for anti-CCP antibodies (Table 3). In contrast, levels of sLepR were lower with greater BMI (Fig. 1 and Table 3).

**Table 3:** sLep, sLepR and respective fat mass ratios of RA patients according to BMI and anti-CCP status

	Normal weight			Preobese			Obese		
	BMI 18.50–24.99 kg/m^2^			BMI 25.00–29.99 kg/m^2^			BMI ≥30.00 kg/m^2^		
	*Anti-CCP*								
Measurement	Negative	Positive	*P*	Negative	Positive	*P*	Negative	Positive	*P*
	n = 6	n = 15		n = 8	n = 13		n = 4	n = 18	
sLep, ng/Ml	51.09 ± 29.76	43.93 ± 44.54	0.267	66.90 ± 51.78	155.34 ± 118.56	0.104	104.04 ± 29.21	206.91 ± 101.91	0.033
sLepR, ng/Ml	60.46 ± 14.20	59.82 ± 21.13	0.898	56.47 ± 13.79	48.49 ± 11.55	0.518	45.89 ± 4.63	35.39 ± 9.55	0.101
sLep/sLepR, ng/Ml	0.89 ± .49	1.02 ± 1.69	0.244	1.36 ± 1.43	3.05 ± 2.75	0.240	2.13 ± 0.86	6.55 ± 3.43	0.048
sLep/fat mass, ng/mL/kg	3.44 ± 1.72	2.86 ± 2.08	0.564	2.73 ± 2.30	6.39 ± 4.98	0.053	3.27 ± 0.87	5.77 ± 2.73	0.033
sLepR/fat mass, ng/mL/kg	4.11 ± 1.01	4.78 ± 2.79	0.925	2.25 ± 0.65	1.99 ± 0.43	0.529	1.45 ± 0.27	1.01 ± 0.39	0.101

Continuous variables are represented as mean ± standard deviation; the Mann-Whitney *U* test was used to compare differences between groups. *BMI* body bas index, *anti-CCP* anti-cyclic citrullinated peptide antibodies, *sLep* serum leptin, *sLepR* soluble leptin receptor)

sLep, sLepR and sLep/sLepR were strongly correlated with fat mass, BMI, insulin and HOMA-IR, as is shown in Table 4. Levels of sLep were positively correlated with ESR (Table 4). There was no correlation between sLep or sLepR and other parameters including anti-CCP antibodies. Notwithstanding, analysis of partial correlation showed that anti-CCP antibodies were correlated with sLep/fat mass ratio (partial *r* = 0.347, *P* = 0.033) after adjustment for age, S4ST and fat mass.

**Table 4:** Correlation between anti-CCP, leptin, sLepR, or leptin/sLep, and adiposity, inflammatory and metabolic markers

	Anti-CCP	sLep	sLepR	sLep/sLepR
Measurement	*R*	*r*	*r*	*r*
	*P*	*P*	*P*	*P*
BMI, kg/m^2^	−0.154	0.494	−0.472	0.556
	0.221	<0.001	<0.001	<0.001
Body fat mass, %	−0.174	0.570	−0.458	0.587
	0.166	<0.001	<0.001	<0.001
ESR, mm/h	0.137	0.257	−0.259	0.355
	0.294	0.033	0.059	<0.001
CRP, mg/L	0.293	0.063	−0.177	0.092
	0.021	0.597	0.205	0.515
sLep, ng/Ml	−0.004	-	−0.557	0.940
	0.977		<0.001	<0.001
sLepR, ng/Ml	−0.134	0.613	-	−0.740
	0.354	<0.001		<0.001
Insulin μUI/Ml	−0.083	0.427	−0.148	0.314
	0.567	0.001	0.286	<0.001
HOMA-IR	−0.091	0.416	−0.072	0.236
	0.533	0.002	0.607	0.092

Pearson *r* coefficient was calculated. *Anti-CCP* anti cyclic citrullinated peptide antibodies, *BMI* body mass index, *ESR* erythrocyte sedimentation rate, *CRP* C-reactive protein, *DAS-28* disease activity index in 28 joints, *sLepR* soluble leptin receptor, *FM* fat mass, *HOMA-IR*, homeostatic model assessment-insulin resistance. *RF* rheumatoid factor

### sLep and sLepR quartiles

When we classified sLep level by quartiles, we observed increasing ESR, body fat ratio, HOMA-IR and decreasing sLepR level. When we grouped the sLepR level by quartiles, we observed increasing ESR, body fat ratio, DAS28 ESR and DAS28 CRP (Table 5).

**Table 5:** Adiposity, metabolic and inflammation markers in RA patients grouped by sLep and sLepR quartiles

sLep ng/Ml	Quartile 1	Quartile 2	Quartile 3	Quartile 4
	<36.27	36.27–72.75	72.76 –177.48	>177.48
	n = 14	n = 16	n = 17	n = 17
Body fat ratio, kg/m^2^	5.92 ± 2.42	8.34 ± 2.90	11.16 ± 2.58	13.82 ± 3.43
ESR, mm/h	18.37 ± 10.53	19.37 ± 11.76	20.17 ± 13.14	27.17 ± 13.65
HOMA-IR	2.23 ± 2.11	3.52 ± 3.98	3.4 ± 2.55	8.25 ± 13.32
sLepR, ng/Ml	69.30 ± 17.62	48.24 ± 7.50	47.21 ± 11.48	36.48 ± 12.49
sLepR ng/Ml	Quartile 4	Quartile 3	Quartile 2	Quartile 1
	>59.35	48.74–59.34	38.83–48.73	<38.83
	n = 11	n = 14	n = 11	n = 15
Body fat ratio, kg/m^2^	6.59 ± 2.63	8.77 ± 2.56	9.30 ± 4.49	11.59 ± 4.73
ESR, mm/h	15.91 ± 9.26	18.14 ± 10.52	19.50 ± 9.90	27.94 ± 12.64
DAS-28 (ESR)	3.08 ± 1.46	3.09 ± 1.25	3.74 ± 1.05	4.02 ± 1.45
DAS-28 (CRP)	2.86 ± 1.37	2.82 ± 1.19	3.39 ± 1.06	3.64 ± 1.43

*FMI* fat mass index, *ESR* erythrocyte sedimentation rate, *HOMA-IR* homeostatic model assessment insulin resistance, *sLepR* soluble leptin receptor, *DAS-28* disease activity score in 28 joints, *CRP* C-reactive protein

## Discussion

In this study, we observed greater sLep levels and sLep/sLepR ratio in RA patients compared to age-, gender- and BMI-matched controls (Table 1); these associations were more evident in RA patients who were overweight or obese (Table 2). After normalization of sLep with fat mass (sLep/fat mass ratio), patients with RA also had greater values of sLep (nanograms per milliliter) per kilogram of fat mass in every BMI category (Table 1). Considering this, patients with RA have greater leptin resistance than that observed in obese controls [36, 37]. sLep, sLep/sLepR ratio and sLep/fat mass ratio in serum from obese RA patients was higher according to positivity for anti-CCP antibodies. Agrawal et al., found that leptin induced the release of IL-6, TNF-α and IL-10 by B lymphocytes, high levels of leptin with high levels of these proinflammatory cytokines, and a chronic inflammatory environment [38]. In RA proinflammatory cytokines like TNF-α and IL-6 might also increase leptin production by adipocytes, leading to chronic hyperleptinemia and leptin resistance [39].

This is the first report showing increased leptin production in association with anti-CCP status in preobese and obese RA patients (Fig. 1a, 1d). This suggests that leptin may be an important mediator for sustaining autoimmune humoral responses in RA, especially in anti-CCP positive patients. Leptin has a myriad of effects triggering or perpetuating acute and chronic inflammation, locally or systemically [40]. Leptin has a role in innate and adaptive immunity, acting as an anti-inflammatory and proinflammatory mediator. Several studies have been performed to investigate the signaling pathways at the molecular level, to address the pleiotropic activity of leptin. Leptin is able to trigger the phosphorylation of different pathways involving extracellular-signal-regulated kinases (ERK)-1, ERK-2, p38 mitogen-activated protein kinase (MAPK), and phosphoinositide 3-kinase (PI3K) in peripheral blood mononuclear cells (PBMCs) eliciting T cell proliferation. Dendritic cell differentiation is favored by STAT3 phosphorylation induced by leptin [41, 42]. In adaptive immunity, leptin incentivizes T cell differentiation towards T helper (Th)1 and Th17 effector cells, activation of B cells, and inhibition of T regulatory (T_reg_) cell proliferation [43].

Leptin blockade in experimental autoimmune encephalomyelitis (EAE) improves clinical score, disease progression and relapses by inhibition of T cell proliferation and cytokine secretion toward a Th2/regulatory profile [44]. In a report of Amarlyo et al. [45] leptin levels were increased in lupus patients, and in murine models leptin promotes the survival and proliferation of autoreactive T cells, explained by upregulation of B cell lymphoma 2 protein (Bcl-2).

Some previous reports on leptin in RA did not report correlation with clinical and inflammatory markers like CRP or ESR. The inability to reproduce these findings may be because most of the reported studies did not include enough overweight or obese patients and did not considered the fat mas distribution or the sLep/fat mass ratio [46–48].

In addition to the leptin resistance state observed in our obese RA patients, we observed an association but no correlation between anti-CCP antibodies and increased sLep, sLep/sLepR and sLep/fat mass ratios according to BMI (Fig. 1a, c, d). Notwithstanding, analysis of partial correlation showed that after controlling for age, fat mass and subcutaneous fat (S4ST) there was an association between serum titers of anti-CCP and sLep (partial *r* = 0.347, *P* = 0.033).

Anti-CCP antibodies are highly specific for RA. Published data suggest distinct pathogenic mechanisms underlying anti-CCP-positive RA [49]. RA patients positive for anti-CCP antibodies have more aggressive disease with early radiographic changes [49, 50]. In the context of a proinflammatory state or autoimmune condition such as RA, leptin could be a stimulator of B cells and plasma cells to produce higher titers of anti-CCP antibodies [51].

After classifying patients by sLep and sLepR quartiles we observed, a trend for higher ESR, and CRP-based and ESR-based DAS-28 with greater sLep and lower sLepR levels. The former observations suggest that other molecular pathways/molecules might be involved in the regulation of leptin in RA but also the possible involvement of leptin in the loss of immune tolerance towards citrullinated proteins in RA. We suggest that leptin could be a surrogate marker of severity or chronicity of humoral immunity in RA in the presence of obesity.

Despite the intensive study of leptin in RA, there are no reports on the association between sLep and anti-CCP antibodies. We consider this study is relevant to open new research fields to allow further examination of the role of these molecules in the pathogenesis of RA. In addition, it is important to evaluate the activity of enzymes such as PADI4 and Dickkopf-1 (DKK-1) in order to improve the characterization of the leptin pathway in RA.

## Conclusions

In preobese and obese RA patients there is increased production of sLep according to anti-CCP positivity. This phenomenon suggests there is an additive effect of chronic inflammation resulting from RA and obesity in which leptin favors the humoral response against citrullinated proteins. In summary, the data observed in our study suggest sLep could be a surrogate marker of chronicity and humoral immunity in RA in the presence of obesity.

## Abbreviations

ACR: American College of Rheumatology
ANOVA: one-way analysis of variance
Anti-CCP: anti-cyclic citrullinated peptide antibodies
Bcl-2: B-cell lymphoma 2 protein
BFR: body fat ratio
BMI: body mass index
CRP: C-reactive protein
CVD: cardiovascular disease
DAS28: Disease activity score in 28 joints
EAE: experimental autoimmune encephalomyelitis
ELISA: enzyme-linked immunosorbent assay
ERK: extracellular-signal-regulated kinases
ESR: erythrocyte sedimentation rate
HOMA-IR: homeostasis model assessment-insulin resistance
IL: interleukin
IR: insulin resistance
IRB: Institutional Review Board
JAK: janus kinase
kDa: kiloDaltons
MAPK: mitogen-activated protein kinases
mTOR: mammalian target of rapamycine
NF: tumor necrosis factor
PI3K: phosphoinositide 3-kinase
RA: rheumatoid arthritis
RF: rheumatoid factor
S4ST: sSum of four skinfold thicknesses
SAD: sagittal abdominal diameter
sLep: serum leptin
sLepR: serum leptin receptor
STAT3: Signal transducer and activator of transcription 3
TCR: T cell receptors
Th: T helper
VFA: visceral fat area
WHO: World Health Organization
WHR: waist to hip ratio

## References

[1] Smolen JS, Aletaha D (2015). Rheumatoid arthritis therapy reappraisal: strategies, opportunities and challenges. Nat Rev Rheumatol..

[2] Wasko MC, Kay J, Hsia EC, Rahman MU (2011). Diabetes mellitus and insulin resistance in patients with rheumatoid arthritis: risk reduction in a chronic inflammatory disease. Arthritis Care Res..

[3] Versini M, Jeandel PY, Rosenthal E, Shoenfeld Y (2014). Obesity in autoimmune diseases: not a passive bystander. Autoimmun Rev..

[4] Rosen ED, Spiegelman BM (2006). Adipocytes as regulators of energy balance and glucose homeostasis. Nature..

[5] MacDougald OA, Hwang CS, Fan H, Lane MD (1995). Regulated expression of the obese gene product (leptin) in white adipose tissue and 3 T3-L1 adipocytes. Proc Natl Acad Sci USA.

[6] Maffei M, Halaas J, Ravussin E, Pratley RE, Lee GH, Zhang Y (1995). Leptin levels in human and rodent: measurement of plasma leptin and ob RNA in obese and weight-reduced subjects. Nat Med..

[7] Tian G, Liang JN, Wang ZY, Zhou D (2014). Emerging role of leptin in rheumatoid arthritis. Clin Exp Immunol..

[8] Ge H, Huang L, Pourbahrami T, Li C (2002). Generation of soluble leptin receptor by ectodomain shedding of membrane-spanning receptors in vitro and in vivo. J Biol Chem..

[9] Huang L, Wang Z, Li C (2001). Modulation of circulating leptin levels by its soluble receptor. J Biol Chem..

[10] Liu C, Liu XJ, Barry G, Ling N, Maki RA, De Souza EB (1997). Expression and characterization of a putative high affinity human soluble leptin receptor. Endocrinology..

[11] Zhang J, Scarpace PJ (2009). The soluble leptin receptor neutralizes leptin-mediated STAT3 signalling and anorexic responses in vivo. Br J Pharmacol..

[12] Lou PH, Yang G, Huang L, Cui Y, Pourbahrami T, Radda GK (2010). Reduced body weight and increased energy expenditure in transgenic mice over-expressing soluble leptin receptor. PLoS One..

[13] Sandhofer A, Laimer M, Ebenbichler CF, Kaser S, Paulweber B, Patsch JR (2003). Soluble leptin receptor and soluble receptor-bound fraction of leptin in the metabolic syndrome. Obes Res..

[14] Laimer M, Ebenbichler CF, Kaser S, Sandhofer A, Weiss H, Nehoda H (2002). Weight loss increases soluble leptin receptor levels and the soluble receptor bound fraction of leptin. Obes Res..

[15] Zastrow O, Seidel B, Kiess W, Thiery J, Keller E, Bottner A (2003). The soluble leptin receptor is crucial for leptin action: evidence from clinical and experimental data. Int J Obes Relat Metab Disord..

[16] Knobelspies H, Zeidler J, Hekerman P, Bamberg-Lemper S, Becker W (2010). Mechanism of attenuation of leptin signaling under chronic ligand stimulation. BMC Biochem..

[17] Pal R, Sahu A (2003). Leptin signaling in the hypothalamus during chronic central leptin infusion. Endocrinology..

[18] Salazar-Paramo M, Gonzalez-Ortiz M, Gonzalez-Lopez L, Sanchez-Ortiz A, Valera-Gonzalez IC, Martinez-Abundis E (2001). Serum leptin levels in patients with rheumatoid arthritis. J Clin Rheumatol..

[19] Targonska-Stepniak B, Majdan M, Dryglewska M (2008). Leptin serum levels in rheumatoid arthritis patients: relation to disease duration and activity. Rheumatol Int..

[20] Seven A, Guzel S, Aslan M, Hamuryudan V (2009). Serum and synovial fluid leptin levels and markers of inflammation in rheumatoid arthritis. Rheumatol Int..

[21] Bokarewa M, Bokarew D, Hultgren O, Tarkowski A (2003). Leptin consumption in the inflamed joints of patients with rheumatoid arthritis. Ann Rheum Dis..

[22] Tong KM, Shieh DC, Chen CP, Tzeng CY, Wang SP, Huang KC (2008). Leptin induces IL-8 expression via leptin receptor, IRS-1, PI3K, Akt cascade and promotion of NF-kappaB/p300 binding in human synovial fibroblasts. Cell Signal..

[23] Muraoka S, Kusunoki N, Takahashi H, Tsuchiya K, Kawai S (2013). Leptin stimulates interleukin-6 production via janus kinase 2/signal transducer and activator of transcription 3 in rheumatoid synovial fibroblasts. Clin Exp Rheumatol..

[24] Meyer M, Sellam J, Fellahi S, Kotti S, Bastard JP, Meyer O (2013). Serum level of adiponectin is a surrogate independent biomarker of radiographic disease progression in early rheumatoid arthritis: results from the ESPOIR cohort. Arthritis Res Ther..

[25] Kang Y, Park HJ, Kang MI, Lee HS, Lee SW, Lee SK (2013). Adipokines, inflammation, insulin resistance, and carotid atherosclerosis in patients with rheumatoid arthritis. Arthritis Res Ther..

[26] Olama SM, Senna MK, Elarman M (2012). Synovial/serum leptin ratio in rheumatoid arthritis: the association with activity and erosion. Rheumatol Int..

[27] Xibille-Friedmann D, Bustos-Bahena C, Hernandez-Gongora S, Burgos-Vargas R, Montiel-Hernandez JL (2010). Two-year follow-up of plasma leptin and other cytokines in patients with rheumatoid arthritis. Ann Rheum Dis..

[28] Arnett FC, Edworthy SM, Bloch DA, McShane DJ, Fries JF, Cooper NS (1988). The American Rheumatism Association 1987 revised criteria for the classification of rheumatoid arthritis. Arthritis Rheum..

[29] WHO. Obesity: preventing and managing the global epidemic. Report of a WHO Consultation. WHO Technical Report Series 894. Geneva: World Health Organization, 2000. 11234459

[30] Durnin JV, Rahaman MM.The assessment of the amount of fat in the human body from measurements of skinfold thickness. 1967. Br J Nutr. 2003 Jan 89;1:147–55. 12572562

[31] Nagai M, Komiya H, Mori Y, Ohta T, Kasahara Y, Ikeda Y (2008). Development of a new method for estimating visceral fat area with multi-frequency bioelectrical impedance. Tohoku J Exp Med..

[32] Prevoo ML, van ‘t Hof MA, Kuper HH, van Leeuwen MA, van de Putte LB, van Riel PL (1995). Modified disease activity scores that include twenty-eight-joint counts. Development and validation in a prospective longitudinal study of patients with rheumatoid arthritis. Arthritis Rheum.

[33] Wintrobe MM, Landsberg JW. A standardized technique for the blood sedimentation test. 1935. Am J Med Sci. 2013 Aug 346;2:148–53. 10.1097/MAJ.0b013e31826caf12 23689045

[34] Levy JC, Matthews DR, Hermans MP (1998). Correct homeostasis model assessment (HOMA) evaluation uses the computer program. Diabetes Care..

[35] Stern SE, Williams K, Ferrannini E, DeFronzo RA, Bogardus C, Stern MP (2005). Identification of individuals with insulin resistance using routine clinical measurements. Diabetes..

[36] Fried SK, Ricci MR, Russell CD, Laferrere B (2000). Regulation of leptin production in humans. J Nutr..

[37] Lee JW, Swick AG, Romsos DR (2003). Leptin constrains phospholipase C-protein kinase C-induced insulin secretion via a phosphatidylinositol 3-kinase-dependent pathway. Exp Biol Med..

[38] Agrawal S, Gollapudi S, Su H, Gupta S (2011). Leptin activates human B cells to secrete TNF-alpha, IL-6, and IL-10 via JAK2/STAT3 and p38MAPK/ERK1/2 signaling pathway. J Clin Immunol..

[39] Lee MJ, Fried SK (2009). Integration of hormonal and nutrient signals that regulate leptin synthesis and secretion. Am J Physiol Endocrinol Metab..

[40] Matarese G, La Cava A, Sanna V, Lord GM, Lechler RI, Fontana S (2002). Balancing susceptibility to infection and autoimmunity: a role for leptin?. Trends Immunol..

[41] Procaccini C, Lourenco EV, Matarese G, La Cava A (2009). Leptin signaling: A key pathway in immune responses. Curr Signal Transduct Ther..

[42] Tian G, Liang JN, Pan HF, Zhou D (2014). Increased leptin levels in patients with rheumatoid arthritis: a meta-analysis. Ir J Med Sci..

[43] Lam QL, Lu L (2007). Role of leptin in immunity. Cell Mol Immunol..

[44] De Rosa V, Procaccini C, La Cava A, Chieffi P, Nicoletti GF, Fontana S (2006). Leptin neutralization interferes with pathogenic T cell autoreactivity in autoimmune encephalomyelitis. J Clin Invest..

[45] Amarilyo G, Iikuni N, Shi FD, Liu A, Matarese G, La Cava A (2013). Leptin promotes lupus T-cell autoimmunity. Clin Immunol..

[46] Toussirot E, Nguyen NU, Dumoulin G, Aubin F, Cedoz JP, Wendling D (2005). Relationship between growth hormone-IGF-I-IGFBP-3 axis and serum leptin levels with bone mass and body composition in patients with rheumatoid arthritis. Rheumatology..

[47] Popa C, Netea MG, Radstake TR, van Riel PL, Barrera P, van der Meer JW (2005). Markers of inflammation are negatively correlated with serum leptin in rheumatoid arthritis. Ann Rheum Dis..

[48] Harle P, Sarzi-Puttini P, Cutolo M, Straub RH (2006). No change of serum levels of leptin and adiponectin during anti-tumour necrosis factor antibody treatment with adalimumab in patients with rheumatoid arthritis. Ann Rheum Dis..

[49] Mouterde G, Lukas C, Goupille P, Flipo RM, Rincheval N, Daures JP (2014). Association of anticyclic citrullinated peptide antibodies and/or rheumatoid factor status and clinical presentation in early arthritis: results from the ESPOIR cohort. J Rheumatol..

[50] van der Helm-van Mil AH, Verpoort KN, Breedveld FC, Toes RE, Huizinga TW (2005). Antibodies to citrullinated proteins and differences in clinical progression of rheumatoid arthritis. Arthritis Res Ther..

[51] Gupta S, Agrawal S, Gollapudi S (2013). Increased activation and cytokine secretion in B cells stimulated with leptin in aged humans. Immun Ageing..

